# Influence of comorbid anxiety and depression disorder on cognition in older adults with epilepsy

**DOI:** 10.3389/fpsyt.2025.1598767

**Published:** 2025-07-31

**Authors:** Hui Qiu, Zongqin Wang, Yanyan Wang

**Affiliations:** ^1^ Department of Psychiatry, Wuhan Mental Health Center, Wuhan, Hubei, China; ^2^ Department of Psychiatry, Wuhan Hospital for Psychotherapy, Wuhan, Hubei, China

**Keywords:** anxiety, depression, cognition, epilepsy, older

## Abstract

**Objective:**

This study aims to investigate the association of comorbid depression and anxiety with cognitive function in older adults with epilepsy.

**Methods:**

A cross-sectional analysis was conducted on 406 older adults (≥65 years) diagnosed with epilepsy between January 2019 and December 2020. Depressive and anxious symptoms were measured using the Hospital Anxiety and Depression Scale (HADS), while cognitive impairment was assessed with the Montreal Cognitive Assessment Test (MoCA). Multivariate linear regression models were used to examine associations between cognitive impairment and anxiety/depression symptoms, adjusting for potential confounders.

**Results:**

Of the 406 adults, 218 (53.7%) showed cognitive impairment. Adults with depression (70.2% vs. 51.0%, *P*<0.01) or anxiety (66.7% vs. 48.8%, *P*<0.01) had a significantly higher prevalence of cognitive impairment compared to those without these conditions. Multivariate linear regression analysis revealed significant associations between cognitive impairment and depression (β=-1.77, 95% CI: -2.67, -0.87; *P*<0.01) and anxiety (β=-2.18, 95% CI: -2.95, -1.42; *P*<0.01).

**Conclusion:**

Anxiety and depression are significantly associated with cognitive impairment in older adults with epilepsy. Early screening and management of these psychiatric conditions are essential to reduce cognitive decline and enhance patient outcomes.

## Introduction

Epilepsy, a prevalent neurological condition affecting around 50 million people worldwide (1), is marked by recurrent seizures and frequently accompanied by psychiatric comorbidities such as anxiety and depression. Research indicates that 30-50% of adults with epilepsy experience clinically significant anxiety or depression, rates substantially higher than in the general population (2).

Cognitive impairment is another common issue in epilepsy, affecting domains like memory, attention, executive function, and language (3). The causes of cognitive decline in epilepsy are multifaceted, involving factors such as underlying brain pathology, recurrent seizures, and the side effects of antiepileptic drugs (AEDs) (4). Recent studies suggest that older adults with epilepsy may exhibit cognitive impairments in certain domains-such as language and executive functions-that are comparable to or even more pronounced than those observed in patients with amnestic mild cognitive impairment (aMCI) (5). In addition, a significant proportion of older patients with epilepsy meet diagnostic criteria for mild cognitive impairment based on standardized neuropsychological criteria (6). Moreover, polytherapy with AEDs and psychiatric symptoms have been independently associated with worse performance in specific cognitive domains such as visuospatial skills and memory (7, 8). Despite this, the specific role of anxiety and depression in exacerbating cognitive deficits, particularly among older adults, remains poorly understood.

Older adults with epilepsy constitute a vulnerable and growing demographic, for whom cognitive impairment can severely impair daily functioning and quality of life (9). This study seeks to examine the impact of comorbid anxiety and depression on cognitive function in older adults with epilepsy, aiming to inform more holistic care strategies.

## Materials and methods

### Study design and participants

This cross-sectional study enrolled older adults (≥65 years) diagnosed with epilepsy at our hospital between January 2019 and December 2020. Epilepsy diagnoses adhered to the International League Against Epilepsy (ILAE) criteria (10), supported by electroencephalogram (EEG) and neuroimaging findings. Exclusion criteria included: 1) concurrent neurological conditions affecting cognition (e.g., Alzheimer’s disease, Parkinson’s disease, or stroke), 2) severe psychiatric disorders (e.g., schizophrenia or bipolar disorder), 3) significant physical illnesses (e.g., heart failure, end-stage renal disease, or diabetes), 4) major sensory or language impairments, and 5) interventions during the study period that could influence cognitive function.

This study was approved by the Ethics Committee of Wuhan Mental Health Center (approval No. 2018025). All procedures involving human participants were performed by the 1964 Declaration of Helsinki and its later amendments or comparable ethical standards. All participants or their caregivers provided informed consent. For adults with cognitive impairment, informed consent was obtained from their guardians, in accordance with the guidelines of the Ethics Committee. All adults or their guardians were fully informed about the purpose, procedures, potential risks, and benefits of the study before agreeing to participation.

### Assessment tools and data collection

The Hospital Anxiety and Depression Scale (HADS) was used to evaluate depressive and anxious symptoms. HADS consists of 14 items, with seven items each for anxiety (HADS-A) and depression (HADS-D). A score of 11 or higher on either subscale indicated clinically significant symptoms (11). The validity and reliability of HADS in older adults aged 65–80 years has been supported by psychometric evaluation in a large general population study (12). Cognitive function was assessed using the Montreal Cognitive Assessment Test (MoCA), a validated screening tool with good sensitivity (83.8%) and specificity (82.5%) for detecting mild cognitive impairment in Chinese older adults, making it appropriate for use in this study (13, 14).

Age, age of first seizure, gender, education level, marital status, neurological etiology, seizure type, seizure focus, seizure frequency, and number of anti-epileptic drugs were collected in medical records. Neurological etiology was determined based on clinical diagnosis and review of neuroimaging results (MRI or CT). Patients were categorized as having either idiopathic epilepsy or secondary epilepsy. Secondary etiologies included stroke, brain tumors, degenerative diseases, brain injury, and other identifiable neurological conditions. Seizure type was classified according to the 2017 International League Against Epilepsy (ILAE) classification system into focal onset and generalized onset seizures. Seizure focus was determined from EEG and imaging findings and categorized as temporal, extratemporal, or unestablished when no clear localization was identified. Seizure frequency was assessed based on self-report and clinical documentation of seizure episodes in the previous 12 months and categorized as “≤5 seizures/month” or “>5 seizures/month.” Number of anti-epileptic drugs (AEDs) was recorded from patients’ medication lists at the time of assessment. Patients receiving only one AED were classified as receiving monotherapy, while those on two or more AEDs were categorized as polytherapy.

### Statistical analysis

Continuous variables with a normal distribution were expressed as mean ± standard deviation (SD) and compared using Student’s t-tests, while non-normally distributed variables were presented as median (interquartile range, IQR) and compared using the Mann–Whitney U test. Categorical variables were expressed as frequencies (%) and compared using Chi-square tests. Multivariate linear regression models were employed to assess the associations between cognitive impairment and anxiety/depression symptoms, adjusting for confounders such as age, age of first seizure, gender, education level, marital status, neurological etiology, seizure type, seizure focus, seizure frequency, and number of anti-epileptic drugs. These variables were selected based on prior evidence indicating their relevance to cognitive outcomes in older adults with epilepsy (15–17). Statistical significance was set at P<0.05. Analyses were performed using SPSS version 22.0 (IBM Corp., Armonk, NY).

A convenience sampling method was employed, enrolling all eligible adults who met the inclusion criteria during the study period. The final sample of 406 adults was considered adequate for multivariate linear regression analyses, with at least 10–15 participants per independent variable, which is a commonly accepted guideline for stable and reliable estimation of regression coefficients.

## Results

A total of 406 older adults with epilepsy were included in the study. Among them, 218 (53.7%) exhibited cognitive impairment, while 188 (46.3%) had normal cognitive function. As shown in **Table 1**, adults with cognitive impairment were significantly older than those without (69.8 vs. 69.1 years, *P*<0.01). 243 (59.9%) were on anti-epileptic drugs (AED) monotherapy and 163 (40.1%) were on polytherapy. The most frequently used AED were levetiracetam (n=182, 44.8%), valproic acid (n=137, 33.7%), carbamazepine (n=94, 23.2%), and lamotrigine (n=61, 15.0%). Among adults with cognitive impairment, a higher proportion received polytherapy compared to those without cognitive impairment (47.7% vs. 34.6%, *P*<0.01). No significant differences were observed in gender, education level, marital status, neurological etiology, seizure type, seizure focus, or seizure frequency between the two groups. However, the cognitive impairment group had significantly higher mean HADS scores for both depression (8.6 vs. 7.7, *P*=0.01) and anxiety (10.4 vs. 8.5, *P*<0.01).

**Table 1:** Participators’ baseline demographic and clinical characteristics .

Variables	Total	Adults with normal cognition	Adults with cognitive impairment	*P* value
N	406	188	218	–
Age, years	69.5 ± 2.7	69.1 ± 2.6	69.8 ± 2.8	<0.01
Age of first seizure, years	49.4 ± 5.0	49.8 ± 4.2	49.0 ± 5.5	0.08
Gender, n (%)				
Male	263 (64.8%)	114 (60.6%)	149 (68.3%)	0.12
Female	143 (35.2%)	74 (39.4%)	69 (31.7%)	
Education^*^, n (%)				
Primary	193 (47.5%)	92 (48.9%)	101 (46.3%)	0.63
Secondary	147 (36.2%)	69 (36.7%)	78 (35.8%)	
Tertiary	66 (16.3%)	27 (14.4%)	39 (17.9%)	
Marital status, n (%)				
Married	282 (69.5%)	137 (72.9%)	145 (66.5%)	0.20
Unmarried	124 (30.5%)	51 (27.1%)	73 (33.5%)	
Neurological etiology, n (%)				
Idiopathic	241 (59.4%)	115 (61.2%)	126 (57.8%)	0.54
Secondary	165 (40.6%)	73 (38.8%)	92 (42.2%)	
Stroke	80 (19.7%)	38 (17.4%)	42 (22.3%)	
Brain tumors	31 (7.6%)	13 (6.0%)	18 (9.6%)	
Degenerative diseases	22 (5.4%)	8 (3.7%)	14 (7.4%)	
Brain injury	11 (2.7%)	5 (2.3%)	6 (3.2%)	
Others	21 (5.2%)	9 (4.1%)	12 (6.4%)	
Type of seizures, n (%)				
Focal onset	295 (72.7%)	140 (74.5%)	155 (71.1%)	0.50
Generalized onset	111 (27.3%)	48 (25.5%)	63 (28.9%)	
Epileptic focus, n (%)				
Temporal	202 (49.8%)	100 (53.2%)	102 (46.8%)	0.23
Extratemporal	130 (32.0%)	60 (31.9%)	70 (32.1%)	
Unestablished	74 (18.2%)	28 (14.9%)	46 (21.1%)	
Number of seizures per month, n (%)				
≤5	194 (47.8%)	93 (49.5%)	101 (46.3%)	0.55
>5	212 (52.2%)	95 (50.5%)	117 (53.7%)	
Number of anti-epileptic drugs, n (%)				
Monotherapy	243 (59.9%)	123 (65.4%)	114 (52.3%)	<0.01
Polytherapy	163 (40.1%)	65 (34.6%)	104 (47.7%)	
HADS-D score	8.2 ± 3.6	7.7 ± 3.3	8.6 ± 3.8	0.01
HADS-A score	9.5 ± 4.8	8.5 ± 4.5	10.4 ± 4.9	<0.01

^*^Primary, elementary/primary school; Secondary, middle and high school; Tertiary, education beyond high school, including university, college, or vocational training.

**Figure 1:**
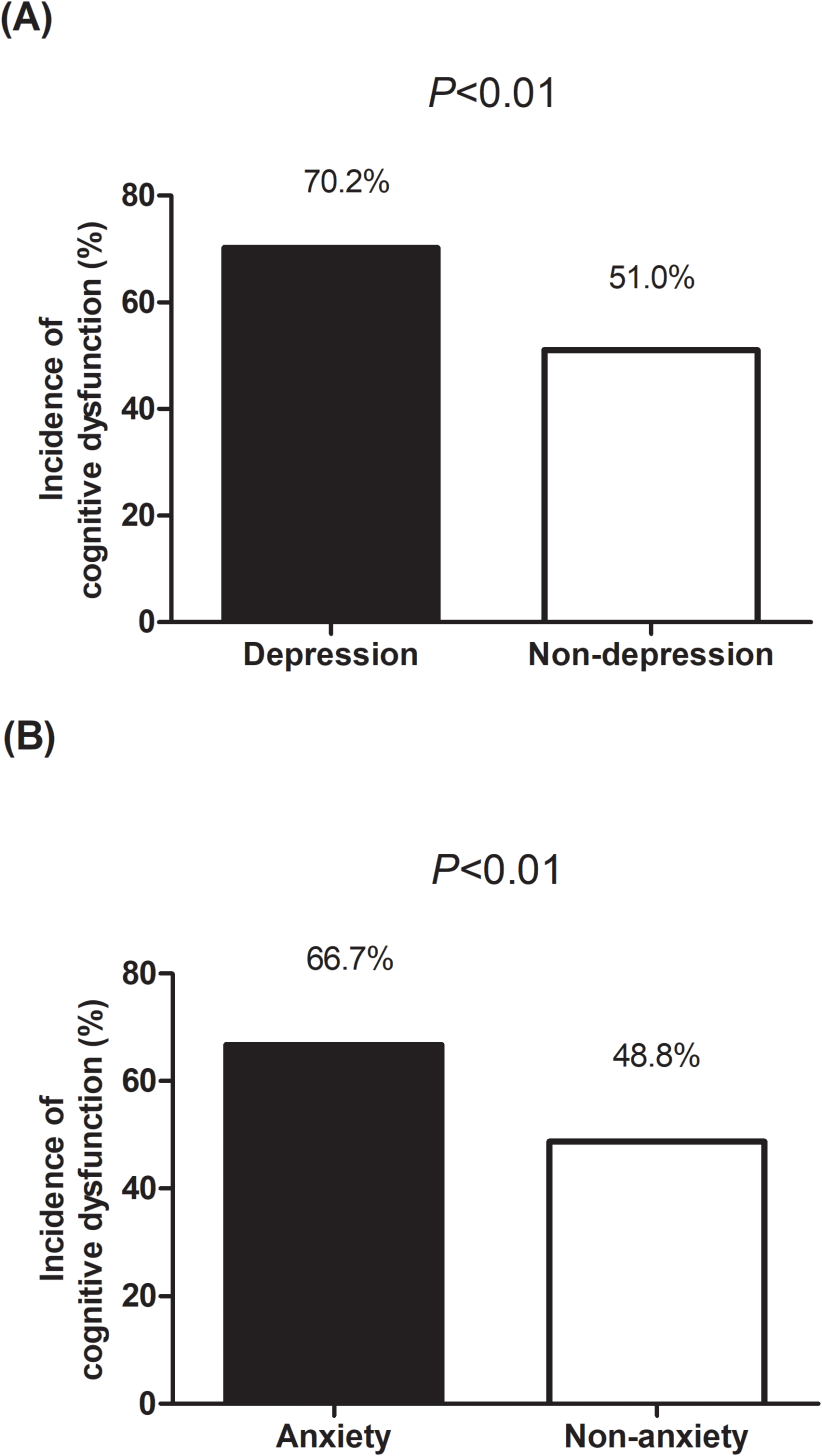
Comparison of the incidence of cognitive impairment among older adults with epilepsy with and without depression **(A)** and anxiety **(B)**.

Adults with depression had a significantly higher prevalence of cognitive impairment compared to those without depression (70.2% vs. 51.0%, *P*<0.01) (**Figure 1**). Similarly, adults with anxiety also showed a higher prevalence of cognitive impairment compared to those without anxiety (66.7% vs. 48.8%, *P*<0.01).

Multivariate linear regression analysis (**Table 2**) revealed significant associations between cognitive impairment and depression (β=-1.77, 95% CI: -2.67, -0.87; *P*<0.01) and anxiety (β=-2.18, 95% CI: -2.95, -1.42; *P*<0.01). Age (β=-0.18, 95% CI: -0.30, -0.07; *P*<0.01), age of first seizure (β=0.09, 95% CI: 0.02, 0.15; *P*<0.01), and anti-epileptic polytherapy (β=-0.71, 95% CI: -1.39, -0.03; *P*=0.04) were also significant predictors of cognitive impairment. Other factors, such as gender, education level, and seizure characteristics, did not show significant associations.

**Table 2:** Multivariate linear regression analysis to explore the association of depression and anxiety with cognitive impairment.

Variables	β	95% CI	*P* value
Depression	-1.77	-2.67, -0.87	<0.01
Anxiety	-2.18	-2.95, -1.42	<0.01
Age	-0.18	-0.30, -0.07	<0.01
Age of first seizure	0.09	0.02, 0.15	<0.01
Male	0.62	-0.03, 1.28	0.06
Education	-0.02	-0.44, 0.40	0.94
Unmarried	0.19	-0.47, 0.86	0.56
Secondary neurological etiology	-0.01	-0.64, 0.62	0.97
Generalized onset	-0.08	-0.76, 0.61	0.83
Extratemporal locus	-0.26	-0.66, 0.15	0.21
Number of seizures per month >5	-0.52	-1.14, 0.10	0.10
Anti-epileptic polytherapy	-0.71	-1.39, -0.03	0.04

## Discussion

The high prevalence of anxiety and depression among older adults with epilepsy is likely driven by the chronicity of the condition, the psychological toll of managing seizures, and the social stigma often associated with epilepsy (18, 19). This study sought to clarify the role of these psychiatric comorbidities in cognitive impairment among older adults with epilepsy. Our results highlight a strong link between anxiety, depression, and cognitive impairment in this population.

Previous studies have reported mixed findings regarding the impact of psychiatric symptoms on cognitive function in epilepsy. Dulay et al. found that depressive symptoms significantly predicted poor performance on memory tests in adults with temporal lobe epilepsy (20). Tang et al. stated that adults with epilepsy who reported more psychological symptoms tended to perform worse in neurocognitive tests (21). In contrast, a study conducted by Monteagudo-Gimeno et al. (22) did not observe a statistically significant association between depression and anxiety and cognitive functions. These discrepancies may stem from differences in study populations, assessment tools, or methodological approaches. Our findings support the notion that anxiety and depression exacerbate cognitive impairment in older adults with epilepsy, underscoring the need for routine screening and early intervention. To support clinical implementation, several pragmatic and validated screening measures are available and can be feasibly applied in diverse settings, including primary care and residential care. For example, Lauderdale et al. provide a comprehensive overview of screening tools designed for older adults that meet key criteria: brief administration time (≤15 minutes), ease of scoring and interpretation (each <5 minutes), and free online availability (23). These tools are designed to identify common psychiatric comorbidities—such as anxiety, depression, suicidal ideation, and substance misuse—at an early stage, thereby facilitating timely intervention. Clinicians are encouraged to incorporate such screening tools into routine epilepsy care for older adults, and to access training resources aimed at improving confidence in mental health assessment in this age group.

The mechanisms underlying the impact of depression and anxiety on cognitive function have been explored in several studies. Chronic anxiety and depression are associated with hypothalamic-pituitary-adrenal (HPA) axis dysregulation, resulting in prolonged elevation of cortisol levels (24, 25). Excessive cortisol has been shown to exert neurotoxic effects on the hippocampus, a brain region critical for learning and memory (26, 27). This can lead to structural atrophy and functional impairments, which may partly explain the increased risk of cognitive deficits observed in adults with comorbid mood disorders (28, 29). Additionally, depression is associated with reduced levels of brain-derived neurotrophic factor (BDNF), which impairs neurogenesis and synaptic plasticity, further compromising cognitive processes (30). Psychosocial factors also play a significant role. Social isolation and reduced physical activity, common in individuals with anxiety and depression, can worsen cognitive decline (31, 32). These factors not only diminish neurogenesis and neurotrophic support but also exacerbate feelings of anxiety and depression, creating a vicious cycle (33). Behavioral factors further contribute to cognitive impairment. Sleep disturbances, often linked to anxiety and depression, negatively affect attention and memory consolidation. Poor medication adherence, another behavioral consequence of these psychiatric conditions, can lead to inadequate epilepsy management and increased seizure frequency, further accelerating cognitive decline (34). Another interesting observation, although not statistically significant, was the higher proportion of participants with a temporal epileptic focus compared to those with extratemporal or unidentified foci. Temporal lobe epilepsy is known to be particularly associated with cognitive deficits, especially in memory domains, due to its close relationship with hippocampal function. While our study did not detect a significant difference among epileptic foci, this trend may still be clinically relevant and warrants further investigation in larger, targeted studies.

Despite the significant findings, this study has several limitations that warrant consideration. First, the cross-sectional design restricts the ability to establish causal relationships. While significant associations between anxiety, depression, and cognitive impairment were observed, the directionality of these relationships remains unclear. Longitudinal studies are necessary to determine causality and elucidate the temporal dynamics between psychiatric symptoms and cognitive decline in older adults with epilepsy. Second, anxiety and depression were assessed using only HADS, a self-reported screening tool. While widely used, HADS primarily captures general emotional distress rather than providing a formal diagnosis of anxiety or depressive disorders. The lack of corroborating assessments—such as clinician-administered interviews or caregiver/family member observations—limits the robustness and diagnostic validity of our findings. Third, cognitive function was evaluated using the MoCA, a brief global screening instrument. Although MoCA is a validated tool for detecting cognitive impairment, it does not provide detailed information about specific cognitive domains or the nature of impairment. The absence of a comprehensive neuropsychological battery limits the depth of interpretation regarding cognitive deficits in this population. Fourth, although several confounders, such as age, gender, epilepsy duration, and antiepileptic drug use, were controlled for, other factors like socioeconomic status, lifestyle habits, and detailed medication adherence were not accounted for. These unmeasured variables could have influenced the observed associations, highlighting the need for more comprehensive data collection in future research. Fifth, the use of convenience sampling may introduce selection bias and limit the generalizability of our findings. Nonetheless, the relatively large sample size and adjustment for multiple confounders enhances the reliability and relevance of our results within the context of a real-world clinical setting. Sixth, there was an imbalance in the gender distribution of our sample, with a higher proportion of male adults. Although sex was included as a covariate in the multivariate analysis and was not significantly associated with cognitive impairment, this discrepancy may limit our ability to explore potential sex-specific differences in the relationship between psychiatric symptoms and cognition.

In summary, this study underscores the substantial impact of comorbid anxiety and depression on cognitive impairment in older adults with epilepsy. Effectively addressing these psychiatric conditions is crucial for improving cognitive outcomes and enhancing the overall quality of life in this vulnerable population.

## Data Availability

The raw data supporting the conclusions of this article will be made available by the authors, without undue reservation.
